# Discoveries Interview: Professor Bhanu P. Jena on the quantum dot-based nanoscale thermometry approach in the detection of pathogens, disease, and life

**DOI:** 10.15190/d.2017.4

**Published:** 2017-05-29

**Keywords:** Quantum dot, thermometry, porosome, muscle efficiency

**Figure 1:**
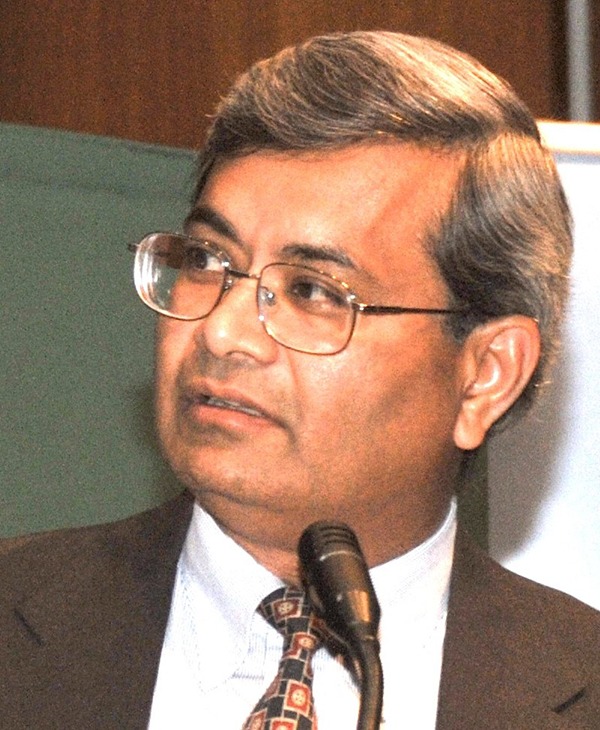
Professor Bhanu P. Jena

**Professor Bhanu P. Jena** “received his PhD degree in Endocrinology, and the Research Excellence Award from Iowa State University in 1988. Following his postdoctoral training at Iowa State and Yale Universities (1989-1994), Prof. Jena joined Yale University School of Medicine as an Assistant Professor, and in 2000, moved to the Department of Physiology at Wayne State University School of Medicine, as Professor, and Founder-Director of the Institute of NanoBioScience, selected in 2006 as one of the top four nano institutes in the US. In 2004, he received the distinction of George E. Palade University Professor and Distinguished Professor. Prof. Jena is the only living University Professor, and the second in Wayne State University’s 160-year history, conferred by the board of governors and the former Wayne State University’s President, Dr. Irvin Reid. At a very early age, Prof. Jena was fascinated by the complexity of the cell, similar to the complexity of a city, yet every aspect of its operation precisely organized and coordinated. His scientific enquiry on cell secretion, which began more than 40 years ago, led to the discovery of the “porosome”, a new cellular structure and the universal secretory portal in cells.

Prof. Jena has received numerous awards and honors for his discoveries, including: election as a Foreign Member of the Georgian National Academy of Science; election as Fellow AAAS; the Swebelius Cancer Research Award, the Hallim Distinguished Award jointly with Prof. Ahmed H. Zewail; Sir. Aaron Klug Award; ASAS Basic Biological Science Award; Ranbaxy Basic Research in Medical Sciences Award; Elected Foreign Member of the Korea Academy of Science & Technology; Elected Foreign Member of the National Academy of Medicine, Romania; George E. Palade Gold Medal, elected to the Academy of Scholars at Wayne State University; six Honorary Doctorates including one from Babes-Bolyai University, Romania, jointly with Professors George E. Palade and Günter Blobel, and Distinguished Visiting Professorships from several academic institutions"^1^.

** *Professor Jena is well known for his pioneering work on the discovery of a new cellular structure -the 'porosome’- the universal secretory machinery in cells, involved in the kiss-and-run fractional release of intra-vesicular contents during secretion^1^.*

## 1. In your recently patented study^2^ you report the determination of muscle efficiency using quantum dots. How does this work?

A thermometer needs to be in direct physical contact with the substrate to make accurate measurements of the temperature at that surface. A cable of some sort such as a peptide antibody, connecting the thermometer to the substrate will not be so precise, hence in our study, we used direct thermometry.

Myosin, a molecular motor that binds and hydrolyses ATP (fuel in living organisms), is required for muscle contraction and movement. Greater heat loss reflects less work performed by the motor, hence decreased efficiency. Two nm cadmium telluride quantum dots (CdTe QDs) fluoresce green, and brighter at lower temperatures, allowing temperature measurements with a spatial and thermal resolution of 80 nm and 1 mK. Using direct association of the 2 nm CdTe QDs to the subject under study, we found rabbit skeletal myosin to be more efficient than bovine cardiac myosin. We further found a gain in efficiency of *Drosophila melanogaster* skeletal muscle overexpressing the PGC-1alpha homolog *spargel*, a known mediator of improved exercise performance in humans^2^.

## 2. This direct thermometry is exciting, yet simple and revealing. Using this form of thermometry, what else is possible?

In our published thermometry study on isolated myosin and fly muscles^2^, we demonstrate that this approach of molecular and cellular calorimetry is able to differentiate between normal and cancerous cells, based on the principle that as a result of increased cellular glycolytic metabolism and higher metabolic rate, cancer cells exhibit higher temperatures^3^, hence their detection as a consequence in loss of fluorescence of the adhered CdTe QDs^2^. Similarly, various metabolic diseases could also be detected using this highly sensitive thermometry approach. Changes in muscle efficiency due to inactivity, in long space flights, and in intensive care unit patients, can also be precisely monitored and managed more effectively. Similarly, we are using this thermometry approach to accurately, rapidly, and cost effectively determine the precise antibiotics required for treatment of patients, as well as to identify the presence of antibiotic resistant bacteria. Again, the principle here is that live bacteria will release heat hence less fluorescence by the adhering CdTe QD’s as opposed to greater fluorescence by dead bacteria. The same principle also applies in detecting life on other planets, with the basic premise that all living organisms have metabolism, and hence they release heat as a consequence. All the above possibilities have been shown in our preliminary studies and are being further pursued more rigorously.

## 3. What advices do you have for young scientists?

Work hard and think out of the box. Child-like curiosity is the best approach to doing science.

## References

[1] (2014). Discoveries Interview: Professor Bhanu P. Jena on the discovery of the porosome, the universal machinery for cellular secretion. Discoveries.

[2] Laha Suvra S, Naik Akshata R, Kuhn Eric R, Alvarez Maysen, Sujkowski Alyson, Wessells Robert J, Jena Bhanu P (2017). Nanothermometry Measure of Muscle Efficiency.. Nano letters.

[3] Stefanadis C, Chrysochoou C, Markou D, Petraki K, Panagiotakos D B, Fasoulakis C, Kyriakidis A, Papadimitriou C, Toutouzas P K (2001). Increased temperature of malignant urinary bladder tumors in vivo: the application of a new method based on a catheter technique.. Journal of clinical oncology : official journal of the American Society of Clinical Oncology.

